# Comparative Analysis of the Self-Propelled Locomotion of a Pitching Airfoil near the Flat and Wavy Ground

**DOI:** 10.3390/biomimetics7040239

**Published:** 2022-12-12

**Authors:** Zhiqiang Xin, Zhiming Cai, Yiming Ren, Huachen Liu

**Affiliations:** The College of Mechanics and Materials, HoHai University, Nanjing 211100, China

**Keywords:** self-propelled locomotion, pitching airfoil, ground effect, wavy ground, vorticity dynamics

## Abstract

In this paper, a pitching airfoil near flat and wavy ground is studied by numerical simulations. The kinematic features of the airfoil and the flow field around it are analyzed to reveal unsteady vorticity dynamics of the self-propelled airfoil in ground effect. The optimal pitching periods at different initial heights above flat ground are obtained, which make the pitching airfoil achieve the maximum lift-to-drag ratio. Compared with flat ground, at the same initial height, the optimal pitching periods vary with the shape of ground. The structure and the strength of the wake vortices shedding from the airfoil are adjusted by the wavelength of ground. This leads to the changes of amplitude and occurrence times of the peak and valley of lift and drag force. The results obtained in this study can provide some inspiration for the design of underwater vehicles in the ground effect.

## 1. Introduction

The phenomenon that flying fish in gliding flight [1,2] and benthic rays [3] achieve excellent locomotion performance by ground effect is ubiquitous in nature. Inspired by this, the ground effect of the airfoil or wing is widely studied [4,5]. The aerodynamic performance is altered when the airfoil or wing moves near the ground.

Current studies on the ground effect mainly focus on the fixed airfoil. Chawla et al. [6] found that the ground effect is displayed significantly only when the height of the airfoil from the ground is less than the average chord length. Ahmed [7] experimentally studied the aerodynamic performance of the airfoil with various ground clearance and angles of attack. The angle of attack has significant influence on the pressure distribution on the lower surface of the airfoil placed in close proximity to a wall. Higher lift force is obtained with decreasing ground clearance only when the angle of attack is in the range of 8 to 10 degrees. In Zerihan and Zhang’s work [8], it was found that when the height of the airfoil from the ground varies from 20% of the chord length to 10%, the downforce increases significantly. However, when the height is less than 10% of the chord length, the downforce declines instead of increasing due to the stall. For the fixed airfoil, the ground proximity has the most significant influence on the lift coefficients. At the same ground proximity, the performance of the fixed airfoil is also affected by other factors such as the angle of attack and airfoil shape.

Birds and aquatic animals can obtain thrust and lift force by flapping their airfoils. When these animals swim or fly near the ground, the unsteady ground effect for the flapping motion is observed unlike the fixed airfoil, because the angle of attack is constantly changing. The physical mechanisms of unsteady ground effect have received widespread attention. An experiment was carried out by Quinn [9] to investigate the unsteady propulsion of the pitching airfoil near the ground. The result shows that the presence of the ground increases the time-averaged thrust and maintains the propulsive efficiency, and the thrust is increased by approximately 40% when the height of the pitching airfoil from the ground is located at an equilibrium point where the mean lift is zero. Mivehchi et al. [10] experimentally studied the rigid flapping airfoil in ground effect and found that the time-averaged mean force alone was not sufficient to fully explain the role of the ground. The distance from the ground has a significant influence on the time-averaged forces and instantaneous forces exerted on the airfoil. In addition, it showed that the strength of the ground effect can be modulated through adjusting propulsive airfoil kinematics without sacrificing thrust. Wu et al. [11] studied a biomimetic energy generator based on the flapping airfoil in ground effect numerically. It was found that a smaller distance from the ground was favorable to generate higher power extraction efficiency for a prescribed amplitude. Zhu et al. [12] reached a similar conclusion and gave an optimum average ground clearance to ensure the highest extraction efficiency. Moreover, the power extraction efficiency of the airfoil can be enhanced obviously by the ground effect while simultaneously increasing the angle of attack and the oscillation frequency of flapping airfoil. Apart from the rigid wing or airfoil in the ground effect, the flexible one in the ground effect was conducted by Wang and Tian [13]. They found that the rigid wall can reduce the generation of lift and drag of the flexible flag and stabilize the flag-fluid system. However, the inertia of flag motion makes the fluid system unstable, and the large inertia leads to chaotic vibration modes, thus reducing the generation of lift and drag. For the flapping airfoil, the oscillation rule and deformation of airfoil play have a significant impact on its ground effect.

The above studies on unsteady ground effects were carried out under the tethered condition and are not sufficient to explain the unsteady ground effect on the locomotion of living animals since those creatures swim or fly freely. During the process of self-propulsion, the moving speed and off-wall distance of self-propelled foil are unknown before the locomotion model of the body has been solved, different from the cases in the tethered condition. Dai [14] and Park [15] studied the effect of self-propelled swimming of a flexible plate near the ground, and the results show that the existence of a wall can improve the cruise speed of self-propelled foil and a suitable flexibility can improve propulsion near the ground. Tang et al. [16] reached a similar conclusion and identified three propulsion regimes for the flapping plate with different heaving frequencies, classified as expensive, benefited, and uninfluenced regimes due to the ground effect. In these studies, an oscillating motion was forced in the leading edge of the flexible plates moving freely. However, Ogunka et al. [17] found that anguilliform swimming near the ground with a ground clearance larger than 0.04 L (fish length) does not improve the swimming performance in a quiescent flow. In nature, there are a wide variety of body shapes and motion characteristics for living animals. The studies about self-propelled locomotion of airfoil or fin in ground effect are still lacking and need further exploration. 

The seabed and the surface of the water are not smooth. Therefore, it is also meaningful to consider the shape variations of the ground, which can investigate the locomotion over real water or ground. The wavy ground effect was studied by Tremblay-Dionne et al. through experiment [18,19]. This is similar to what happens on flat ground, where greater lift is obtained as the ground clearance decreases. Meanwhile, wavy ground with a smaller wavelength and larger amplitude causes more obvious ground effect. In the numerical simulation study of Gao et al. [20] the oscillation frequency and amplitude of the aerodynamic forces were determined mainly by the frequency of the wavy ground and the average ground clearance, respectively. Hu et al. [21] studied the aerodynamic performances of airfoil in wavy ground effect corresponding to different angles of attack. They found that the medium angle of attack was the optimal design cruise angle of attack for a wing-in-ground vehicle. However, these studies only considered the ground effect of fixed airfoil on wavy ground. The explorations of bionic flapping airfoils on wavy ground effect are very few. Therefore, it is important to study the biological locomotion performance over actual uneven terrain.

Much effort has been made to study the steady ground effect and the flapping airfoil under the tethered condition in ground effect. However, a study on the self-propulsion airfoil in ground effect can give a better understanding of the physical mechanisms of birds and benthic fishes moving near the ground, and knowledge about this topic is very limited so far. Therefore, the self-propelled locomotion of a pitching airfoil near flat and wavy ground is investigated using numerical simulation in this study. The vortex dynamics mechanisms of the self-propelled locomotion in ground effect are also analyzed to reveal the relationship of vorticity field and the forces acting on the airfoil and its kinematics characteristic. The systematic analysis of self-propelled airfoil under different motion parameters could contribute to the design and control of ground-effect vehicles.

## 2. Problem Description and Methodology

### 2.1. Problem Description

**Figure 1:**
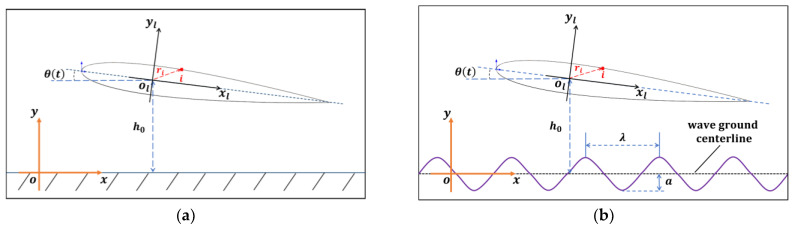
Schematic diagram of the self-propelled airfoil with pitching motion (**a**) Near the flat ground (**b**) Near the wavy ground.

In this study, the airfoil is considered separately from the initial height above flat and wavy ground, and self-propulsion begins according to a given pitching rule. The NACA0012 airfoil with the chord c is used as the shape of the pitching airfoil above the flat and wavy grounds, as shown in Figure 1. Two sets of coordinates are employed in studying the self-propulsion of the airfoil: the local coordinates $\left(x_{l},y_{l}\right)$ and the global laboratory coordinates (x,y). The global laboratory coordinates are fixed on the ground. The origin of the local coordinates is located at the center of mass of the airfoil. The governing equation of pitching motion is given:

(1) $$\theta \left(t\right)=\theta_{0}\sin\left(2\pi ft\right)$$

where θ(t) is the angle of attack, $\theta_{0}$ is the maximum pitching amplitude angle, f is the frequency of pitching, t is the time, h is the height of the airfoil from the ground in Figure 1. A sinusoidal wavy ground is employed in the present study:

(2) $$y=asin\left(\frac{2\pi x}{\lambda }\right)$$

where a and λ are the amplitude and wavelength of wavy ground, respectively.

The four sets of initial height of the airfoil from the ground $h_{0}$, which are $h_{0}=0.5c,\;1.0c,1.5c,2.0c$ and a case without ground effect, are considered in this study. For a certain $h_{0}$, the pitching periods range from 1 s to 5 s.

The hydrodynamic force F and the moment M centered at the COM (center of mass) $o_{l}$, which exerted on the airfoil, are defined as follows [22]:

(3) $$F=-\int_{\partial B}(-pn+\mu \omega \times n)ds$$

(4) $$M=-\int_{\partial B}x\times (-pn+\mu \omega \times n)ds-2\mu \int_{B}\omega_{B}dV$$

where ∂B is body surface, n is the unit outward normal vector of the body surface, $\omega_{B}$ is the angular velocity of the body, ω, p, μ are the fluid vorticity, pressure and dynamic viscosity, respectively.

The governing equation of the locomotion of the two-dimensional airfoil is

(5) $$\{\begin{array}{c} m_{\bar{dt}}^{du_{t}}=F_{d}\\ m_{\bar{dt}}^{dv_{t}}=F_{l}\\ \frac{d}{dt}\left(\omega_{m}\Sigma_{i}m_{i}r_{i}^{2}\right)=M \end{array}$$

where m is the mass of the airfoil, $u_{t}$ and $v_{t}$ are the translational velocity in the x direction and y direction, respectively. $m_{i}$ is the mass of the i element on the airfoil surface, $r_{i}$ is the position vector of the i element (shown in Figure 1). $F_{d}$ and $F_{l}$ are the components of F in the x direction and y direction, respectively. $F_{d}$ is the sum of thrust force and drag force [23]. The unknowns in Equation (5) are the components of translational velocity $u_{t}$, $v_{t}$ and angular velocity $\omega_{m}$. After solving the equations of $u_{t}$, $v_{t}$ and $\omega_{m}$, the change of airfoil displacement and attack angle of the airfoil can be obtained by solving the following equations:

(6) $$\{\begin{array}{c} \frac{dx}{dt}=u_{t}\\ \frac{dy}{dt}=v_{t}\\ \;\;\;\;\frac{d\theta_{m}}{dt}=\omega_{m} \end{array}$$

where $\theta_{m}$ is the angle caused by the external moment, so the attack angle of the airfoil at the next moment is restated as Equation (4) from Equation (1):

(7) $$\theta \left(t\right)=\theta_{m}+\theta_{0}\sin\left(2\pi ft\right)$$

Although the motion velocity of the airfoil changes during the self-propelled process, the motion velocity is close to the initial one. The Reynolds number based on the chord length is Re=ρU¯c/μ=150, where ρ, $\bar{U}$ and μ are the density, the magnitude of motion velocity and dynamic viscosity of fluid, respectively. Thus, the Navier-Stokes equations is solved directly while the Reynolds number in the present study is low. The momentum and continuity equations are expressed as:

(8) $$\frac{\partial u}{\partial t}+\left(u\cdot \nabla \right)u=-\frac{1}{\rho }\nabla p+\frac{\mu }{\rho }\nabla^{2}u$$

(9) ∇⋅u=0

where u is the fluid velocity, ρ is the fluid density, p is the pressure and μ is the dynamic viscosity, respectively.

The numerical simulation of the self-propelled locomotion of the airfoil includes two sets of boundary conditions: the external boundaries of the computational domain and boundary of the airfoil surface.

The external boundary conditions of the computational domain are specified as follows: inflow velocity is 0, so Dirichlet-type boundary condition is used at the inlet boundary as $u_{x}=0$, $u_{y}=0$; at the outlet and top, Neumann-type boundary condition is applied as $\partial u_{x}/\partial n=0$ and $\partial u_{y}/\partial n=0$ to indicate the far field, n is the normal of the boundary; on the bottom, the no-slip boundary condition was specified.

In the initial stage of motion, the flow in the computational domain is at rest. The immersed boundary conditions on the airfoil surface consist of translational velocity and linear velocity produced by rotation.

1. The translational velocity in inertial frame is defined as:

(10) $$V_{t}=u_{t}i+v_{t}j$$

2. The linear velocity generated by the airfoil rotating around the COM $\left(x_{ol},y_{ol}\right)$ in the local rotating frame is defined as:

(11) $$\{\begin{array}{c} u_{r}=-\omega_{m}\;\left(y_{l}-y_{ol}\right)\\ v_{r}=\omega_{m}\;\left(x_{l}-x_{ol}\right)\;\;\; \end{array}$$

where $\left(x_{l},y_{l}\right)$ is the coordinates of the point on airfoil surface, $\omega_{z}$ can be obtained by:

(12) $$\omega_{z}=\dot{\theta }\left(t\right)=\omega_{m}+2\pi f\theta_{0}\cos\left(2\pi ft\right)$$

Thus, the immersed boundary conditions [24] on the airfoil surface can be expressed as follow:

(13) $$\{\begin{array}{c} u_{x}=u_{t}+u_{r}\\ v_{y}=v_{t}+v_{r} \end{array}$$

### 2.2. Numerical Method

In this study, the immersed boundary method is used to handle moving boundary conditions. The immersed boundary method is a technique of defining moving boundary conditions in grids. It was first developed by Peskin [25] to solve heart valve problems. It can be used to simulate the interaction between structure (even the elastic solid) and fluid. The immersed boundary method can deal with the changeable boundaries moving with time on the fixed background mesh, and the mesh does not need to be regenerated with the solid motion. Due to the existence of moving solid, the background mesh is divided into three different types: the mesh element completely belonging to the flow field, the solid mesh element and the immersed boundary (IB) mesh element. The motion problem is solved by repositioning the object in the mesh.

The discrete-direct forcing approach [26,27] is adopted in this paper, which applies the boundary conditions directly on the cells touching the immersed boundary (IB) by modifying the discretized governing equations near the IB. Whether it is the continuous term or the discretized term, no forcing term is introduced into the governing equation, which is different from other indirect imposition approaches. In each time step, the value of the dependent variable in the meshes closely adjacent to the immersed boundary is evaluated by interpolation using the values of neighboring meshes and the boundary condition at the corresponding immersed boundary points [28].

**Figure 2:**
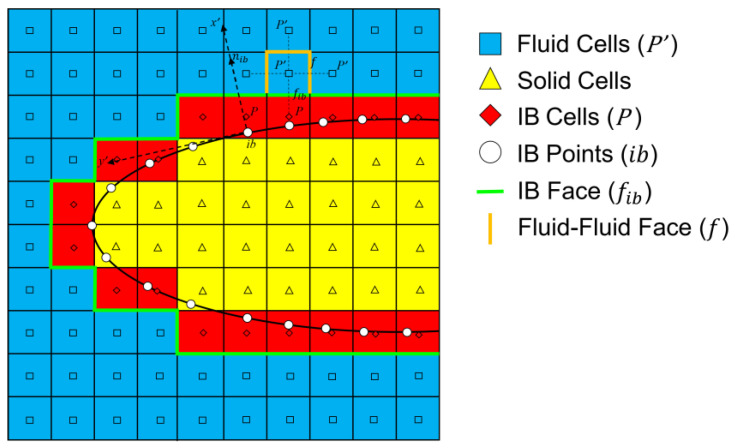
Schematic of computational domain with an immersed boundary.

The polynomial interpolation is used to impose the Dirichlet immersed boundary condition at IB cells, each IB point needs five extended meshes for interpolation in 2D cas-es. Therefore, the interpolation equation is expressed as follows:

(14) $$\phi_{P}=\phi_{ib}+\alpha_{0}\left(x_{P}-x_{ib}\right)+\alpha_{1}\left(y_{P}-y_{ib}\right)+\alpha_{2}\left(x_{P}-x_{ib}\right)\left(y_{P}-y_{ib}\right)\;+\alpha_{3}{\left(x_{P}-x_{ib}\right)}^{2}+\alpha_{4}{\left(y_{P}-y_{ib}\right)}^{2}$$

where x, y are coordinates of computational meshes, and α is unknown coefficients calculated through the weighted least squares method. Subscript P, $P^{\prime }$ and ib represent the IB meshes, fluid meshes and IB points, respectively (the details are shown in Figure 2).

The Neumann immersed boundary condition at IB points is imposed as Equation (15), the interpolation is performed in local coordinate.

(15) $$\phi_{P}=\alpha_{0}+\left[n_{ib}\cdot {\left(\nabla \phi \right)}_{ib}\right]{x^{\prime }}_{P}+\alpha_{1}{y^{\prime }}_{P}+\alpha_{2}{x^{\prime }}_{P}{y^{\prime }}_{P}+\alpha_{3}{\left({x^{\prime }}_{P}\right)}^{2}+\alpha_{4}{\left({y^{\prime }}_{P}\right)}^{2}$$

where n is the normal vector of the interface, the *x′*-axis coincides with the normal to the immersed boundary at the point *ib* for system *x′y′*, and α is the same with which in Equation (14).

The interpolation formula of pressure at IB is defined as follows:

(16) $$\underset{f_{ib}}{\sum }n_{f_{ib}}\cdot u_{f_{ib}}\cdot S_{f_{ib}}=S_{f}\cdot \left(\underset{f}{\sum }n_{f}\cdot \left(\frac{1}{A}\right)\cdot H-\underset{f}{\sum }n_{f}\cdot {\left(\frac{1}{A}\right)}_{f}\cdot {\left(\nabla p\right)}_{f}\right)$$

where subscript $f_{ib}$ denotes the interface between IB meshes and fluid meshes, f denotes the interface of the fluid-fluid mesh near to IB, S is the interface area, u is the velocity on the interface, n is the normal vector of the interface, and A and H are the coefficients matrix which are defined in the literature [28].

For computational domains with a uniform grid around IB, $u_{f_{ib}}$ is calculated by:

(17) $$u_{f_{ib}}=\frac{1}{2}\left(u_{P}+u_{P^{\prime }}\right)$$

The immersed boundary method employed in the present study is implemented based on foam-extend. The governing equations of fluid are solved by the predictor–corrector solver pisoFOAM. The details about discrete-direct IBM approach and pisoFOAM can be referred to the literature [26,27,28].

### 2.3. Numerical Validation

#### 2.3.1. Immersed Boundary Method Validation

In order to verify the immersed boundary method with moving boundary, the flapping motion of an airfoil in ground effect is simulated in this paper. The inflow velocity is U=1.25 m/s, and kinematic viscosity $\upsilon =1\times 10^{-3}{\;m}^{2}∕s$, thus the Reynolds number based on the chord length *c* is Re=150. The governing equation for the airfoil motion can be expressed as $h\left(t\right)=h_{0}+h_{m}cos\left(2\pi ft\right)$ and $\theta \left(t\right)=\theta_{0}sin\left(2\pi ft\right)$. In this subsection, $h_{0}=c,h_{m}=0.4c,\theta_{0}=\pi /4$, and the calculation domain is 5c×3c. The Strouhal number is defined as $St=2h_{0}/\left(T\cdot U\right)$, where T is the pitching period, and the values of St are taken as 0.1, 0.2, 0.3, 0.4, 0.5, respectively. 

**Figure 3:**
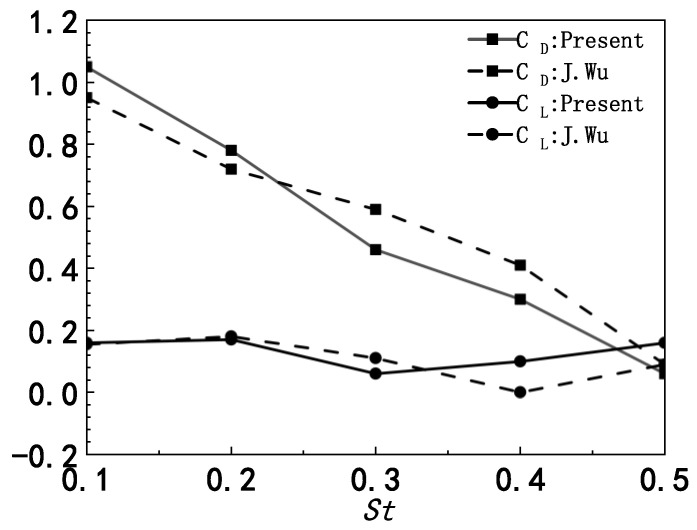
Lift and drag coefficients for different St as $h_{0}=c$.

**Figure 4:**
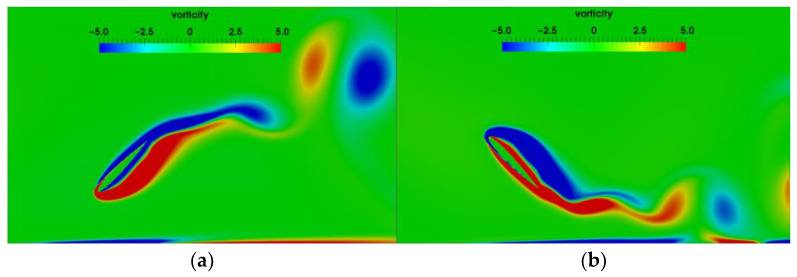
Instantaneous vorticity contours at $h_{0}=c,\;St=0.1$ (**a**) pitching downward (**b**) pitching upward.

Figure 3 presents the lift and drag coefficients of the airfoil at different Strouhal numbers as $h_{0}=c$. The lift and drag coefficients are defined as follows:

(18) $$\{\begin{array}{c} C_{D}=\frac{2F_{D}}{\rho U^{2}c}\\ C_{L}=\frac{2F_{L}}{\rho U^{2}c} \end{array}$$

where $F_{D}$ and $F_{L}$ are the drag and lift force exerted on the airfoil during pitching. When St≤0.2 the lift and drag coefficients are in good agreement with the results of J. Wu [29]. Although some minor difference appears in the cases for high Strouhal numbers, the changing trend is the same. Figure 4 presents the vortex shedding induced by a pitching airfoil. The vortices are regularly detached from the trailing edge of the airfoil. In conclusion, the immersed boundary method that we employ is suitable for this study.

#### 2.3.2. Mesh and Time Step Sensitivity Test

**Figure 5:**
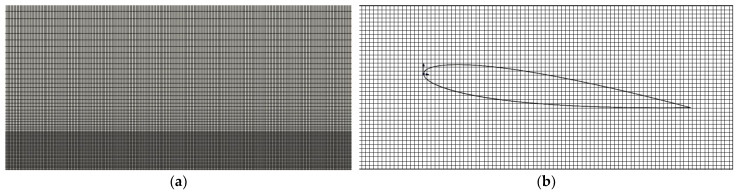
Computational meshes (**a**) full computational domain (**b**) zoomed view of the meshes around the airfoil.

The mesh and time step sensitivity tests are also conducted. The size of the flow field is set as 10c×4c. The initial distance between the center of the airfoil and the ground is $h_{0}=0.5c$, the pitching period is T=1 s, and the pitching amplitude is $\theta_{0}=\pi /12$. Three different meshes and time steps are shown in Table 1. To ensure the accuracy of calculation results and the computing efficiency, the second combination is finally selected. The meshes of full computational domain are shown in Figure 5a, and the meshes in close proximity to the ground are densified. The meshes around the airfoil are shown in Figure 5b.

## 3. Results

The self-propulsion of a pitching airfoil in ground effect are systematically studied using the immersed boundary method in the following sections. The influences of ground clearance, pitching period and pitching amplitude on the self-propelled locomotion of the pitching airfoil in ground effect are comparatively analyzed. The kinematics and mean hydrodynamic force are firstly discussed; and then the corresponding physical mechanisms are revealed by analyzing the relationship of hydrodynamic force and wake vortices and pressure fields.

### 3.1. Unsteady Motion above the Flat Ground

#### 3.1.1. Trajectory

**Figure 6:**
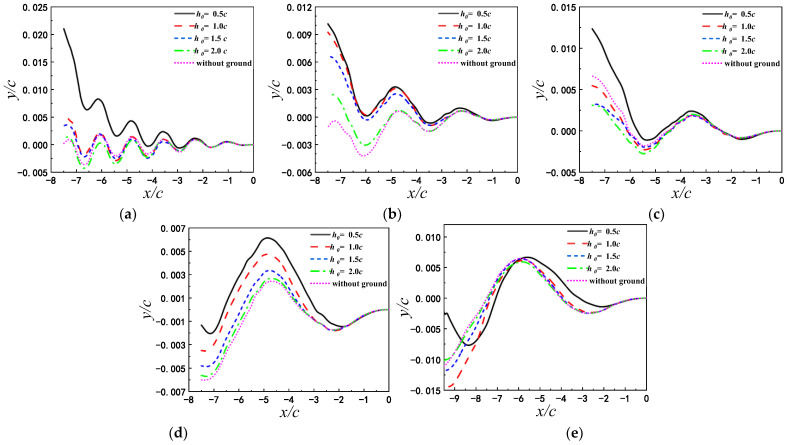
Motion displacements of the airfoil pitching with different $h_{0}$. (**a**) T=1 s  (**b**) T=2 s (**c**) T=3 s (**d**) T=4 s (**e**) T=5 s.

The displacements of the self-propelled airfoil for different $h_{0}$ and T are shown in Figure 6. It can be seen that although the prescribed pitching rule is exerted on the airfoil, the peak of the motion trajectory is constantly changing, which can only be reflected in the self-propelled locomotion. The fluctuation cycles of the motion trajectory are mainly determined by the pitching period of the airfoil. For a certain pitching period T, the ground clearance has a significant effect on the displacement of the self-propelled airfoil. When the pitching period of the airfoil is in the range of 1 s≤T≤4 s (see Figure 6a–d, the mean heights of the airfoil increase with decreasing $h_{0}$. Especially as $h_{0}=0.5c$, the vertical displacement increases most, which is larger than those for other $h_{0}$. On the whole, the influence of ground effect on the motion trajectory of self-propelled airfoil decreases with the increases of $h_{0}$ and T. 

Distinguished from the cases of the high pitching frequency, the displacement of the airfoil exhibits different characteristics in low frequency. For T=5 s, as shown in Figure 6e, the vertical displacement of the airfoil set free from the initial height $h_{0}=0.5c$ becomes less than that of other cases when the airfoil moves to about x=5.5c from the starting point. After x=8.5c, the airfoil corresponding to $h_{0}=0.5c$ starts to move upward and enter the rising stage earlier than that for other $h_{0}$. This shows that when the airfoil moves with a larger pitching period, the ground effect changes the fluctuation period of the motion displacement. In addition, the airfoil with the larger pitching period can first obtain a larger vertical displacement, then drops quickly. For the airfoil with a small pitching period, it cannot rise rapidly, but its displacement shows steady upward tendency. Therefore, for the creatures in nature, the combination of multiple pitching modes is more conducive to survival.

#### 3.1.2. Hydrodynamics

**Figure 7:**
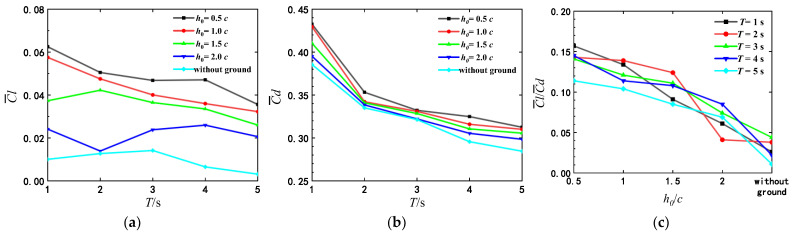
Comparison of mean drag coefficient, mean lift coefficient and lift-to-drag ratio for different pitching periods (**a**)$\;{\bar{C}}_{l}$ (**b**) ${\bar{C}}_{d}$ (**c**) ${\bar{C}}_{l}/{\bar{C}}_{d}$.

From the mean lift and drag coefficients in Figure 7a,b, it can be seen that ${\bar{C}}_{l}$ is always greater than zero, which is consistent with the displacement trend in Figure 6. Through the comparison of the mean drag and lift coefficients for different initial height, the airfoil for the small initial height gains a larger drag and lift. In addition, $C_{d}$ decreases monotonically with pitching period, and $C_{l}$ has the same tendency when the airfoil is in close proximity to the ground ($h_{0}<1.5c$). For the same initial height, the mean drag coefficient is the largest when T=1 s.

Besides $C_{d}$ and $C_{l}$, the lift-to-drag ratio is also the key to analyze the motion performance. Therefore, the lift-to-drag ratio is shown in Figure 7c. It can be found that the lift-to-drag ratios of the airfoil pitching at the same frequency increase with the reducing of $h_{0}$, in the pitching period range considered. When the pitching airfoil is close to the ground, the lift-to-drag ratio increases by 300%. At $h_{0}=0.5c$, the lift-to-drag ratio of the airfoil for T=1 s is the largest, and the lift-to-drag ratios of the airfoil for T=2 s, 3 s, 4 s are close to each other. The airfoil obtains the largest displacement at $h_{0}=0.5c$ and T=1 s as shown in Figure 6. When the airfoil begins to pitch from the same initial height, it is uncertain to improve the lift-to-drag ratio by decreasing the pitching period. It shows that there is the corresponding optimal pitching period at different initial heights. 

### 3.2. Unsteady Motion above the Wavy Ground

#### 3.2.1. Trajectory

In this section, a wavy ground is exerted on the bottom of the computational domain, where the amplitude of the ground a=0.05c, the wavelength is λ=c, 2c, respectively, to obtain different types of wavy ground. It can be seen from the analyses above that the flat ground effect is obvious when $h_{0}=0.5c$. Based on this, only the initial height of the airfoil above the wavy ground center-line $h_{0}=0.5c$ is chosen. According to the results from the literatures [18,19,20], the minimum and maximum lift generally appeared at wave valley and peak of ground, respectively, and they become lager with increasing wave amplitude due to the variation of the effective ground clearance. The influencing mechanism of the wave amplitude on the wavy ground effect are basically clear. Therefore, we focus on the influence of the wavelength and pitching period on the wavy ground effect and the comparison of flat and wavy ground effect in the present study.

**Figure 8:**
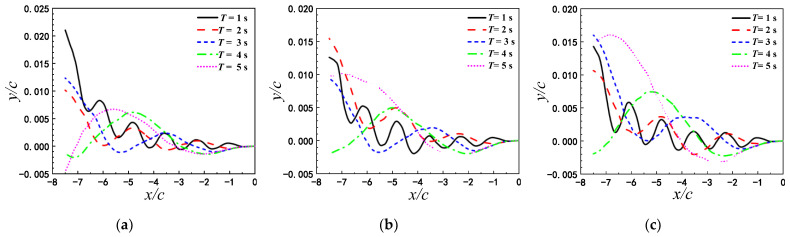
Displacement of the airfoil over flat ground and wavy ground with wavelength parameter λ=2c and λ=c under different pitch periods (**a**) flat ground (**b**) λ=2c  (**c**) λ=c.

The airfoil displacement corresponding to two types of wavy ground and flat ground are given in Figure 8. Regardless of flat or wavy ground, the pitching airfoil can obtain the higher vertical displacement at T=1 s. Compared with the flat ground, airfoils pitching with appropriate periods also enhance the higher vertical displacement in wavy ground effect, except the case of T=1 s. For the wavy ground with wavelength λ=2c, the maximum final displacement can be obtained is at T=1 s or T=2 s; For wavy ground with wavelength λ=c, the maximum final displacement can be obtained is at T=1 s or T=3 s. 

#### 3.2.2. Hydrodynamics

**Figure 9:**
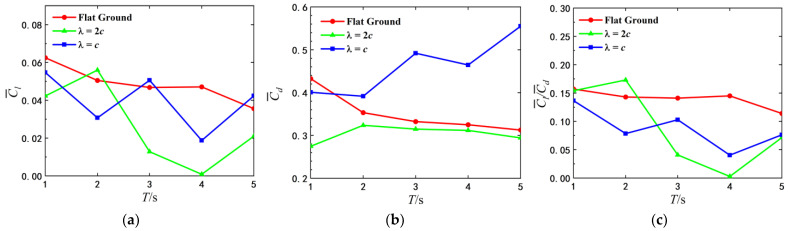
Average lift and drag coefficients and average lift drag ratio of airfoil under different pitching periods.(**a**) ${\bar{C}}_{l}$ (**b**) ${\bar{C}}_{d}$ (**c**) ${\bar{C}}_{l}/{\bar{C}}_{d}$.

In addition to the displacement of the airf oil, the hydrodynamics should also be analyzed to reveal the displacement variation. In Figure 9, the average lift coefficient, drag coefficient and lift-drag ratio of airfoil over two different wavy ground and the flat ground are compared at $h_{0}=0.5c$. Compared with the flat ground, the lift and drag of airfoil in wavy ground effect are more sensitive to the change of pitch period. The optimal pitching periods for different ground conditions are not identical when the initial height is constant. At λ=2c, the airfoil with the small pitch period obtains a larger lift. The average lift coefficient of the airfoil pitching with T=2 s is the largest, even higher than that of the airfoil over the flat ground. Since the average drag coefficient varies very little with the pitch period, the lift-drag ratio is also the largest as T=2 s. And it can be seen from Figure 8 that the final vertical displacement of the airfoil with T=2 s is the maximum. Therefore, as the wavelength of the wave ground λ=2c and the initial height of the airfoil $h_{0}=0.5c$, the optimal pitch period of the airfoil is T=2 s.

As λ=c, the lift and drag coefficients of airfoil fluctuate with the increase of flapping period. The average lift coefficient of the airfoil pitching with T=1 s is the largest. This explains the reasons that the airfoil pitching with T=1 s has a relatively large final vertical displacement, as shown in Figure 8. Therefore, when the wavelength of the wave ground λ=c and the initial height of the airfoil h=0.5c, the optimal pitch period of the airfoil is T=1 s.

### 3.3. Mechanism of Ground Effect on the Self-Propelled Airfoil

To delve into the fluid physical mechanism of self-propelled locomotion of the pitching airfoil in ground effect, the relationship of lift and drag coefficients of airfoil and the vorticity dynamics for the flat ground and wavy ground are investigated in the following.

#### 3.3.1. Vortex Structure and Its Effect Near the Flat Ground

**Figure 10:**
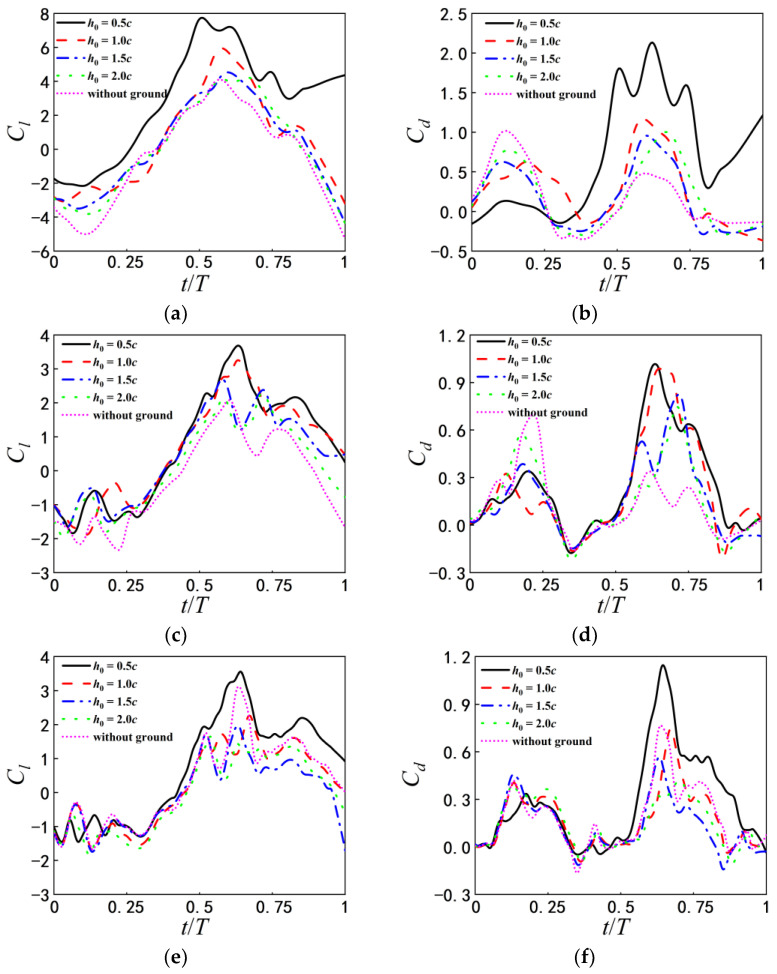

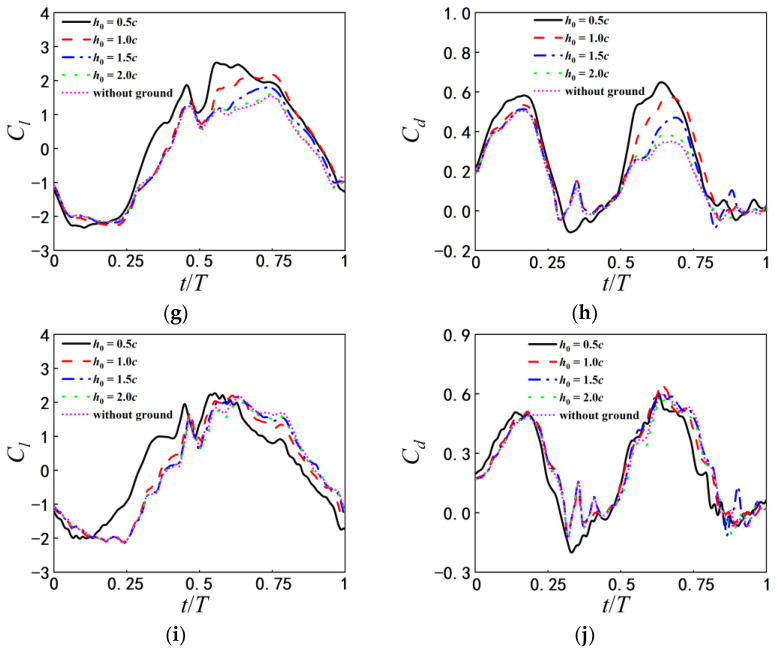
Time histories of $C_{l},\;C_{d}$ corresponding to different initial heights (**a**) $C_{l}$ at T=1 s (**b**) $C_{d}$ at T=1 s (**c**) $C_{l}$ at T=2 s (**d**) $C_{d}$ at T=2 s  (**e**) $C_{l}$ at T=3 s (**f**) $C_{d}$ at T=3 s (**g**) $C_{l}$ at T=4 s (**h**) $C_{d}$ at T=4 s  (**i**) $C_{l}$ at T=5 s (**j**) $C_{d}$ at T=5 s.

The time histories of lift and drag coefficients of airfoil in one flapping cycle for different initial heights above the flat ground are shown in Figure 10. In general, the lift and the drag coefficient fluctuate around zero, and the amplitude of the peak value is greater than that of the valley value. The lift and drag coefficients of airfoil depend on $h_{0}$ and T together. For the same pitching period, the peak value of lift and drag vary with ground proximity. While the pitching periods are relatively larger, the ground proximity has less effect on the lift and drag coefficients of airfoil.

Figure 10a,b shows the lift and drag coefficients as T=1 s. The lift coefficient at $h_{0}=0.5c$ is always significantly higher than those of $h_{0}>0.5c$, and the drag coefficient becomes higher only when *t*/*T* = 0.25–1.0 s. The obvious increase of the drag coefficients is closely related to the change of mean attack angle induced by the ground effect during the process of self-propelled locomotion. The increase of mean attack angle leads to the expansion of the longitudinal area. For a pitching airfoil under the tethered condition, the lift and drag coefficients repeat completely in a period, because the change of attack angle is prescribed periodically. 

With the increase of pitching period, the influence of the initial height $h_{0}$ on the lift and drag coefficients gradually weakens. In addition, in the case of the large pitching period and the smaller initial height, the drag coefficients have two peaks in one flapping cycle. Figure 10i,j shows the lift and drag coefficients for different initial heights as the pitching period T=5 s. Through the analysis of Figure 6e, it is concluded that when the pitching period is T=5 s, the vertical increment of displacement of the airfoil for the initial height $h_{0}=0.5c$ is not the maximum in the whole locomotion process, which corresponds to the change in the lift coefficient in Figure 10i. In the first half period, the lift coefficient at $h_{0}=0.5c$ increases fast and becomes greater than that of the case with $h_{0}>0.5c$; in the second half period, the lift coefficient decreases much and becomes less than other cases.

**Figure 11:**
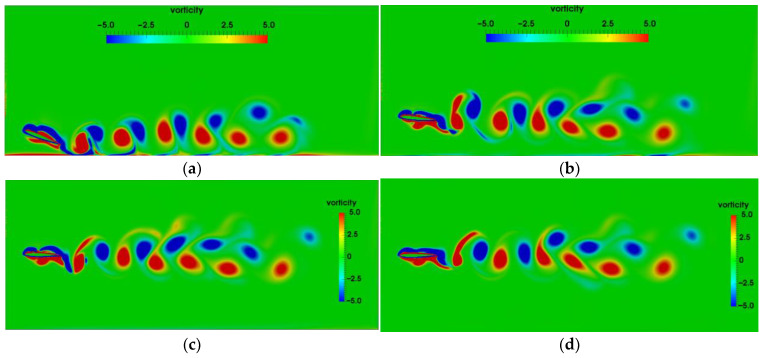
Vorticity contours as pitching period T=1 s (**a**) $h_{0}=0.5c$ (**b**) $h_{0}=1.0c\;$ (**c**) $h_{0}=2.0c$ (**d**) Without ground.

Figure 11 presents the vortex distributions around the flapping airfoil with different $h_{0}$ at T=1 s. As $h_{0}=0.5c$, the attack angle of the airfoil is significantly greater than that for other initial heights. The counter-rotating vortices induced by the vortices shedding from on the surface of the airfoil appear on the ground. The closer the airfoil is to the ground, the higher the strength of the vortices generated on the ground is.

The positive and negative vortex core in the wake behind the airfoil form vortex pairings (dipoles). The wake vortices are up-tilted due to the wall confinement, which is the same as Dai’s results [14]. The ground effects are correspondingly enhanced. As the initial height increase to $h_{0}=1.0c$, the effect of ground on the wake vortices becomes weaker. When the initial height becomes lager than 2.0c, the motion of the airfoil and vortex shedding are hardly affected by the ground, basically similar to the airfoil without ground effect.

**Figure 12:**
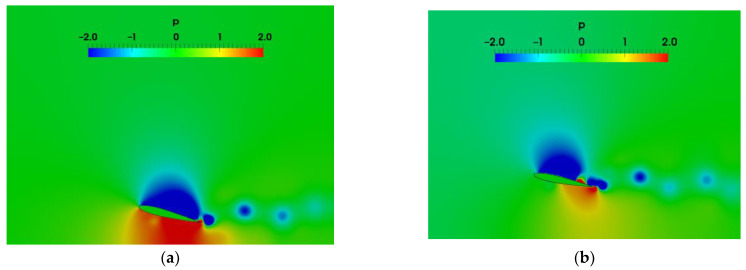
Pressure distribution around the airfoil as the pitching period T=1 s (**a**) $h_{0}=0.5c$ (**b**) $h_{0}=1.0c$.

In order to further reveal the physical mechanism reasons that the airfoil closer to the ground obtains a higher lift force, Figure 12 presents the pressure distribution around the airfoil induced by the vortex shedding at the lift peak moment. It can be found that the pressure differences between the upper and lower surfaces of the airfoil corresponding to $h_{0}=0.5c$ are significantly greater than that of the airfoil corresponding to $h_{0}=1.0c$. Thus, the airfoil corresponding to $h_{0}=0.5c$ acquires higher lift forces.

**Figure 13:**
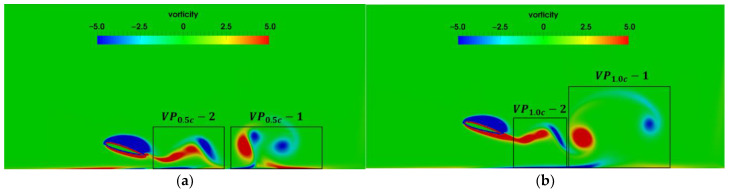
Vorticity contour as the pitching period T=5s (**a**) $h_{0}=0.5c$ (**b**) $h_{0}=1.0c$.

Figure 13 shows the vorticity contours for the initial heights of $h_{0}=0.5c$ and $h_{0}=1.0c$ as the pitching period is T=5 s. In Figure 13, $VP_{0.5c}$ and $VP_{1.0c}$ denote the vortex pairing corresponding to $h_{0}=0.5c$ and $h_{0}=1.0c$, respectively, 1 and 2 denote the appearance sequence of the vortex pairing. Compared with the wake vortices for T=1 s, the vortices near the trailing edge become slenderer and tend to distribute along the horizontal direction, and moreover, the transverse vortex spacing is larger. The strength of the wake vortices for T=5 s is less than that for T=1 s. As a result, the lift and drag coefficients for T=5 s are relatively lower. Figure 13a shows that for the airfoil as the initial height $h_{0}=0.5c$, there is significant mutual induction of the vortex shedding form the airfoil and the secondary vortices attached to the ground. However, for the airfoil at $h_{0}=1.0c$ as shown in Figure 13b, the effect of ground on vortex shedding is obviously weakened, and no vortex pairing with high strength is formed. As T=5 s, the transverse vortex spacing increases with the ground proximity.

#### 3.3.2. Evolution of the Wake Vortices and Its Effect Near the Wavy Ground

**Figure 14:**
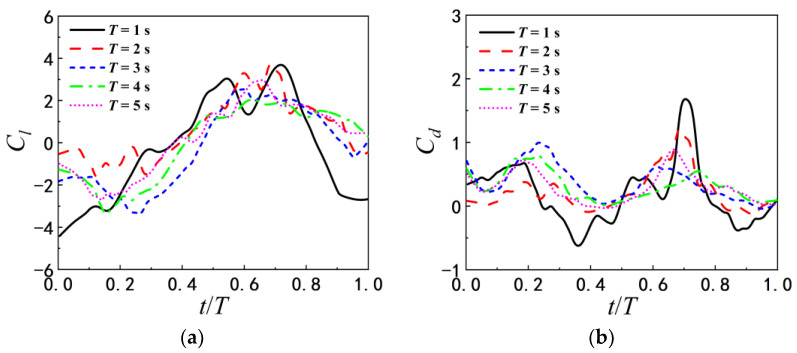

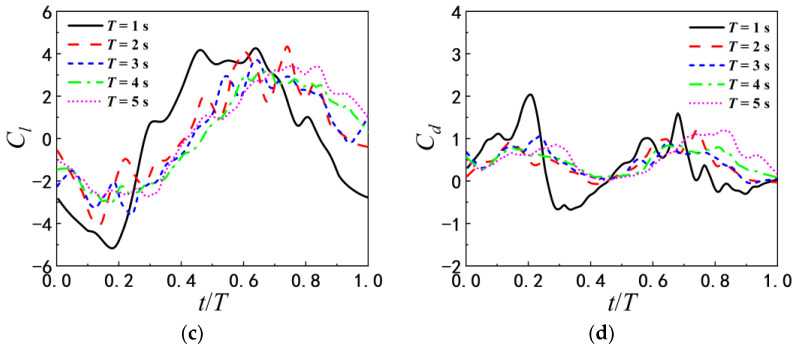
Time histories of lift and drag coefficients of the airfoil in one period under different pitching periods (**a**) $C_{l}$ as λ=2c (**b**) $\;C_{d}$ as λ=2c (**c**) $C_{l}$ as λ=c (**d**) $C_{d}$ as λ=c.

The influences of wavy ground are explained in detail by the combinatorial analysis of the locomotion of airfoil and the evolution of the wake vortices in this subsection. Figure 14 shows the time histories of lift and drag coefficients of airfoil with different pitching periods above the wavy ground within a period. The amplitudes of the maximum and minimum lift and drag coefficients of airfoil pitching with T=1 s are larger than those for other pitching period. However, the lift coefficients of airfoil pitching with pitching with T=1 s are not always the highest over a pitching period, different from the flat ground. The occurrence times of the peak and valley of lift and drag coefficients mainly depend on the pitching period T, but are slightly adjusted by the wave length of ground. As λ=2c, there are a main peak and a secondary peak in the curve of lift coefficient for T=1 s and T=2 s, and the drag coefficients $C_{d}$ for all pitching periods have two peaks in one pitch period, which occur at t=0.2 T and t=0.7 T respectively. As λ=c, the variation trend of lift and drag coefficients is roughly the same as that of λ=2c. For the same pitching period, the amplitudes and the occurrence times of the peak and valley of lift and drag coefficients change with the wave length of ground.

**Figure 15:**
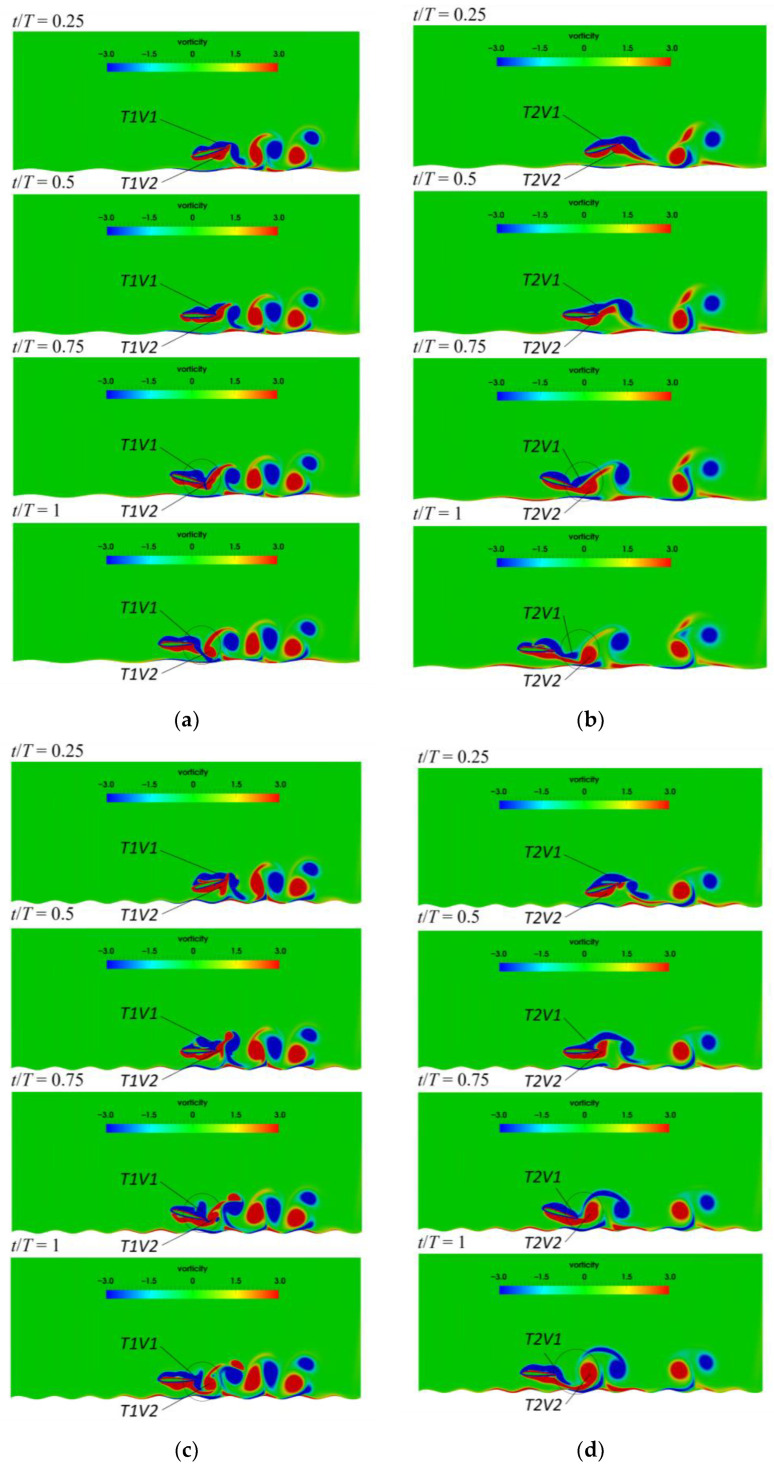
Vorticity contours in a pitching period (**a**) T=1 s, λ=2c (**b**) T=2 s, λ=2c (**c**) T=1 s, λ=c (**d**) T=2 s, λ=c.

The vorticity contours at four different time instant in one flapping cycle are presented to illustrate the process of vortex shedding, as shown in Figure 15. T1 and T2 indicate the pitching period corresponding to T=1 s and T=2 s, respectively, and V1 and V2 denotes the number of wake vortex in Figure 15. When 0.5<t/T<0.75, the airfoil pitches downward, and the airfoil pitch upward when 0<t/T<0.25 or 0.75<t/T<1.0. As T=1 s and λ=2c, the positive vortex T1V2 is generated on the lower surface of airfoil, and the negative vortex T1V1 is formed on the upper surface of airfoil at t/T=0.25. T1V2 begins to shed off from the trailing edge at t/T=0.5. At t/T=0.75, the vortex T1V1 moves to the trailing edge, and the vortex T1V1 and vortex T1V2 begin to interact significantly. The vortex T1V2 still does not shed off completely when vortex T1V1 and vortex T1V2 interact at the trailing edge of the airfoil. Therefore, the flow near the trailing edge is complex. When 0.5<t/T<0.75, the angle of attack is positive, corresponding to the peak value of the lift coefficient. During this time, the airfoil just moves over the peak of the ground, so that the lift coefficient is enhanced by the wavy ground, as shown in Figure 14. The results of Lee et al. [30] show that the maximum and the minimum lift coefficient of the fixed airfoil over the wavy ground appeared at the crest and the trough of ground, respectively. As the analysis above, the higher lift force can be obtained but only when the pitching airfoil with the positive angle of attack just moves the crest of the wavy ground. At t/T=1, the vortex T1V1 begins to shed from the upper surface of the trailing edge and partially sheds into the wake. Then the pitching motion enters the next cycle.

Combined with the influence of wavy ground, the wake vortices for different pitching periods exhibit different characteristics, which cause the variations of the force acting on the airfoil. As T=2 s, the pitching frequency is slow, so the vortices generated at the previous time have moved away from the trailing edge. The transverse distance between the wake vortices becomes larger with the pitching period. Thus, the influences of the wake vortices on the hydrodynamic force exerted on the airfoil gradually weakened with the increase of the pitching period. As the wavelength of ground is reduced to λ=c, the feature of the vortex shedding during the self-propelled locomotion process of the airfoil and the transverse distance between the wake vortices are similar to λ=2c. So the variation trend and amplitude of lift and drag coefficients are also roughly the same as those of λ=2c. However, for λ=c, the ground shape changes obviously along the swimming direction. Thus, the structure of the vortex cores is correspondingly adjusted, and the strength of the vortex cores increase slightly. This leads to the changes of the amplitudes and the occurrence times of the peak and valley of lift and drag force.

**Figure 16:**
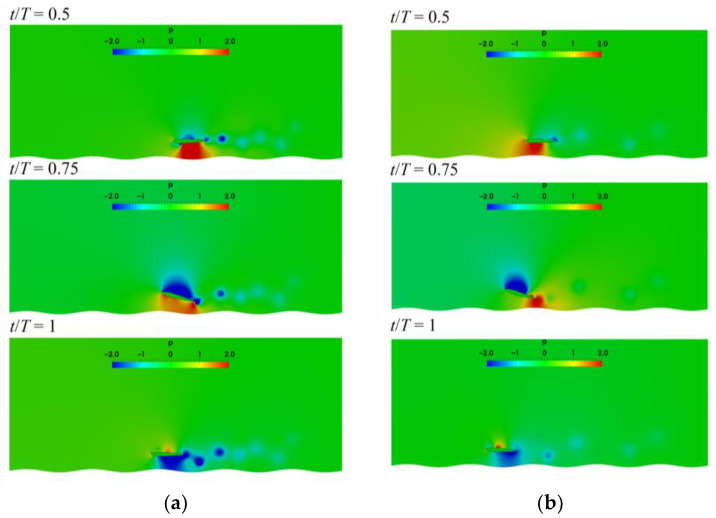

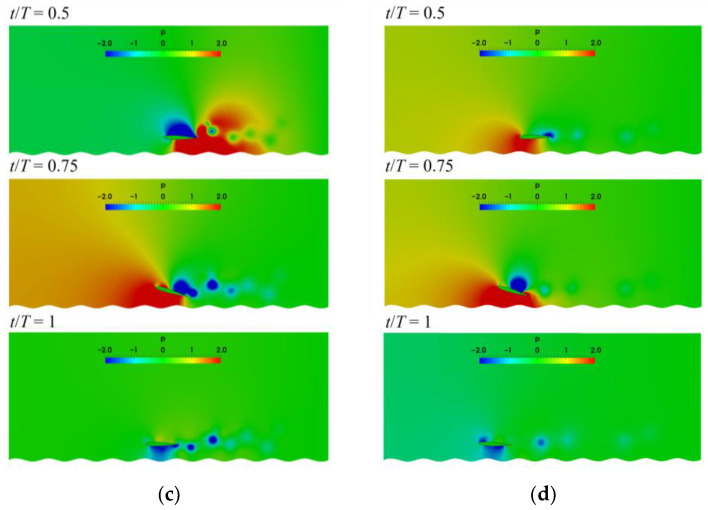
Pressure distribution (**a**) T=1 s, λ=2c  (**b**) T=2 s, λ=2c (**c**) T=1 s, λ=c (**d**) T=2 s, λ=c.

The pressure fields induced by the wake vortices are shown in Figure 16, in order to reveal the physical mechanism of the lift and drag coefficients in Figure 14. As λ=2c,at t/T=0.5 and t/T=0.75, the positive pressure on the lower surface of airfoil with T=1 s is larger than that of airfoil with T=2 s. The pressure difference between the upper and lower surfaces at this time causes the larger lift coefficient in Figure 14. When t/T=1, the greater negative pressure appears on the lower surface of the airfoil with T=1 s, leading to the small lift coefficient. As the wavelength of ground becomes λ=c, it can be seen that the pressure on the lower surface of the airfoil with T=1 s is also larger than that with T=2 s during t/T=0.5 and t/T=0.75. Therefore, regardless of the wavelength of ground, the airfoil with T=1 s can obtain a larger instantaneous lift, but the drag of the airfoil is also large in this case. The pressure difference between the upper and lower surfaces of the airfoil over smaller wavelengths of ground are relatively larger, and therefore the amplitude of lift and drag coefficients are amplified. The occurrence time of the maximum pressure difference between the upper and lower surfaces of the airfoil changes with the pitching period and the ground wavelength. 

## 4. Conclusions

The fluid mechanisms of self-propelled locomotion of the pitching airfoil over the flat and wavy ground are investigated by immersed boundary methods in the present study. The influence of the initial height from the ground, pitching period and shape of the ground on self-propelled locomotion of the pitching airfoil in ground effect and the corresponding vorticity dynamic mechanism are obtained. The main conclusions are as follows:

1. When the airfoil pitching with a smaller period is in close proximity to the flat ground, the airfoil can obtain a larger lift and lift-to-drag ratio due to the ground effect. Thus, the mean vertical displacement of the pitching airfoil increases with decreasing $h_{0}$. With the increase of pitching period, the influence of the ground proximity on the lift and drag coefficients gradually weakens. That’s because that the wake vortices near the trailing edge of the airfoil pitching with a larger period become slenderer, and moreover, the transverse vortex spacing is larger. By comparing the lift-to-drag ratios for different *T* and $h_{0}$, it is found that there are the corresponding optimal pitching periods for the pitching airfoil at different initial heights.
2. Regardless of the flat or wavy ground, the pitching airfoil can obtain the larger lift and higher vertical displacement at T=1 s and $h_{0}=0.5c$. However, compared with the flat ground, the airfoils pitching with other appropriate periods also achieve the higher vertical displacement in wavy ground effect. At the same initial height, the optimal pitching periods for different ground conditions are not identical. The occurrence times and the amplitudes of the peak and valley of lift and drag coefficients mainly depend on the pitching period T. But the structure of the vortex cores is correspondingly adjusted by the wave length of ground and the strength of the vortex cores increase slightly with decreasing the ground wavelength. This leads to the changes of the amplitudes and the occurrence times of the peak and valley of lift and drag force.

In general, in order to obtain the optimal locomotion performance, it is necessary to choose the appropriate pitching parameters according to the ground proximity and the type of the ground. The combination of multiple pitching modes inspired from swimming near the ground is more conducive for man-made underwater vehicle.

## Figures and Tables

**Table 1:** Mesh and time step sensitivity tests.

Number of Grids	Δx/c	Δt(s)	${\bar{C}}_{d}$	${\bar{C}}_{l}$
50,000	0.02	0.01	0.2923	0.6925
150,000	0.01	0.005	0.2224	0.3580
250,000	0.005	0.005	0.2287	0.3473
250,000	0.005	0.0025	0.2275	0.3466

## Data Availability

Not applicable.
